# Targeted disruption of the mouse *Csrp2 *gene encoding the cysteine- and glycine-rich LIM domain protein CRP2 result in subtle alteration of cardiac ultrastructure

**DOI:** 10.1186/1471-213X-8-80

**Published:** 2008-08-19

**Authors:** Julia F Sagave, Markus Moser, Elisabeth Ehler, Sabine Weiskirchen, Doris Stoll, Kalle Günther, Reinhard Büttner, Ralf Weiskirchen

**Affiliations:** 1 Institute of Clinical Chemistry and Pathobiochemistry, RWTH- University Hospital Aachen, Germany; 2 Max Planck-Institute for Biochemistry, Martinsried, Germany; 3 The Randall Division of Cell and Molecular Biophysics and The Cardiovascular Division, King's College London, UK; 4 Qiagen, Hilden, Germany; 5 Institute of Pathology, University Hospital Bonn, Germany

## Abstract

**Background:**

The cysteine and glycine rich protein 2 (CRP2) encoded by the *Csrp2 *gene is a LIM domain protein expressed in the vascular system, particularly in smooth muscle cells. It exhibits a bimodal subcellular distribution, accumulating at actin-based filaments in the cytosol and in the nucleus. In order to analyze the function of CRP2 *in vivo*, we disrupted the *Csrp2 *gene in mice and analysed the resulting phenotype.

**Results:**

A ~17.3 kbp fragment of the murine *Csrp2 *gene containing exon 3 through 6 was isolated. Using this construct we confirmed the recently determined chromosomal localization (Chromosome 10, best fit location between markers D10Mit203 proximal and D10Mit150 central). A gene disruption cassette was cloned into exon 4 and a mouse strain lacking functional *Csrp2 *was generated. Mice lacking CRP2 are viable and fertile and have no obvious deficits in reproduction and survival. However, detailed histological and electron microscopic studies reveal that CRP2-deficient mice have subtle alterations in their cardiac ultrastructure. In these mice, the cardiomyocytes display a slight increase in their thickness, indicating moderate hypertrophy at the cellular level. Although the expression of several intercalated disc-associated proteins such as β-catenin, N-RAP and connexin-43 were not affected in these mice, the distribution of respective proteins was changed within heart tissue.

**Conclusion:**

We conclude that the lack of CRP2 is associated with alterations in cardiomyocyte thickness and hypertrophy.

## Background

In vertebrates, the cysteine- and glycine-rich proteins (CRPs) encoded by the *Csrp *genes are evolutionarily conserved proteins that define a subset of zinc-binding LIM domain proteins. As structural hallmarks, these proteins exhibit two LIM domains with a characteristic spacing, adjacent glycine-rich repeats, and a potential nuclear localization signal [1]. Originally, this family of LIM domain proteins included three members (CRP1, CRP2, CRP3/MLP) that were independently isolated in the course of different experimental strategies [2-4]. Subsequently, based on structural and sequence similarities, the thymus LIM protein (TLP) was grouped into this subclass of LIM domain proteins [5]. The four CRPs possess significant differences in their temporal and spatial patterns of expression raising interesting questions regarding the physiological and biological significance of the CRP multigene family [6]. For example, it is uncertain if these proteins perform unique functions or substitute for each other within a living organism. The cell types and organs that express the different CRPs suggest several hypothetical functions for this group of LIM domain proteins, including possible roles in organization and stabilization of the contractile myofibrillar/cytoskeletal apparatus [6-8], maintenance of cellular functions [3], differentiation [5], transcriptional regulation [9], and in the establishment of fibrogenic responses [10]. In addition to this potential functional versatility, there is growing evidence supporting the notion that the two LIM domains of CRPs serve as protein interfaces mediating specific protein-protein interactions thereby arranging two or more protein constituents into nuclear transcription or cytoskeletal complexes [11,12]. In this regard, the CRP3 protein (also termed MLP for muscle LIM protein) is best characterized. It is a positive regulator of myogenic differentiation that was first identified in a screen for genes that become transcriptionally upregulated as a result of skeletal muscle denervation [4]. In accordance, the overexpression of MLP in C2 myoblasts potentiates myogenic differentiation [4] and the absence of the *Csrp3/Mlp *gene product causes a phenotype of dilated cardiomyopathy underscoring the hypothesis that CRP3/MLP is an essential regulator of cardiac muscle development [7]. In line with this hypothesis, the morphological and clinical picture of dilated cardiomyopathy in humans is associated with altered *Csrp3/Mlp *expression [13] and *Csrp3/Mlp *mutations were found in families suffering from dilated as well as from hypertrophic cardiomyopathy [14,15].

Together, CRP1 and CRP2 were shown to be potent smooth muscle differentiation cofactors triggering the conversion of pluripotent 10T1/2 fibroblasts into smooth muscle cells when overexpressed together with serum response factor (SRF) and GATA proteins [9]. Compatible with this presumed function is the finding that CRP2 is present at highest levels in arterial samples [16,6]. Moreover, a recent report demonstrated that CRP2 can effectively switch on smooth muscle gene activity in adult cardiac myocytes [17] suggesting that CRP2 has essential functions in controlling smooth muscle gene activity.

Furthermore, during embryogenesis and in adult tissue, *Csrp2 *gene expression is also prominently associated with mesenchyme and epithelia [18,19]. Interestingly, compared to other CRP family members, CRP2 expression begins early in gestation and has a distinct pattern of tissue distribution during cardiovascular development. [18]. CRP2 is expressed transiently in early embryonic cardiomyocytes similar to smooth muscle cell markers like α-smooth muscle actin, calponin, and SM22α [18] but its expression is downregulated in adult cardiomyocytes. Additionally, it was demonstrated that the expression of CRP2 is downregulated with cellular dedifferentiation induced by oncogenic transformation, injury, or wound healing [3,16,10].

Recently, it was demonstrated that the loss of CRP2 did not result in any apparent gross vascular defects or altered expression of smooth muscle cell markers [20]. Moreover, vascular development, morphology, cell proliferation, endothelial regeneration and the expression of several characteristic smooth muscle specific genes were similar between WT and *Csrp2 *nulls. However, the loss of CRP2 is correlated with increased neointima formation in response to vascular injury. Furthermore, vascular smooth muscle cells isolated from mice lacking CRP2 migrated more rapidly in response to PDGF-BB with an increased activation of the Rho GTPase Rac1 suggesting that *Csrp2 *and its protein product CRP2 are functionally linked to cell migration [20].

We here report about the generation and characterization of a similar *Csrp2 *null mouse model. We demonstrate that these deficient mice are viable and fertile, exhibiting a mild cardiac phenotype in which the cardiomyocytes display a slight increase in their thickness, indicating moderate hypertrophy at the cellular level. In line with these findings, the peculiarity of heart architecture reflected by the typical arrangement of intercalated disc-associated proteins (i.e. β-catenin, N-RAP, connexin-43) was altered suggesting that CRP2 is involved in the organization of the cytoskeleton in cardiac muscle cells.

## Results

### Chromosomal localization of the murine *Csrp2 *gene

**Figure 1:**
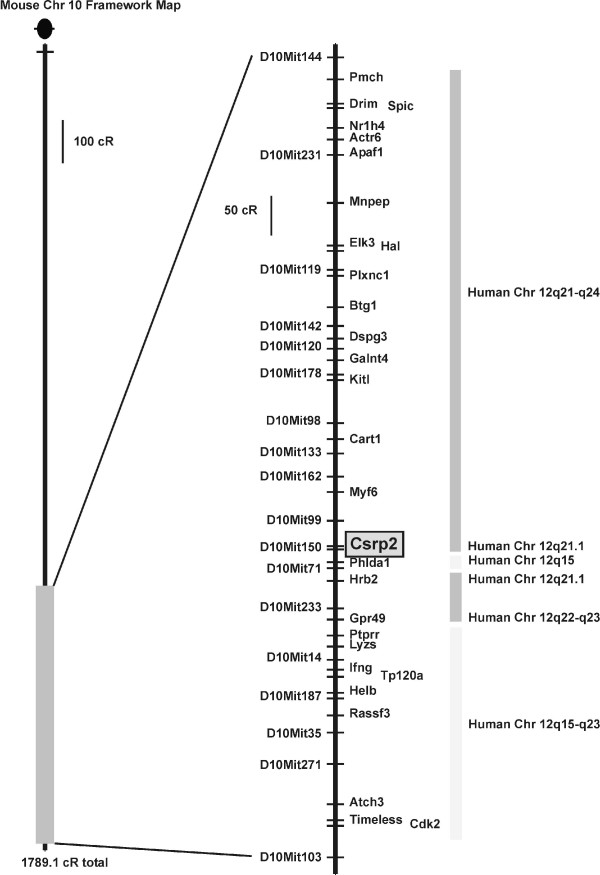
**Assignment and map location of murine *Csrp2 *on Chromosome 10**. We typed the 100 cell hybrid DNAs (1–100) of the mouse T31 whole-genome-radiation hybrid panel and two sets of independent A23 hamster (H) and 129 mouse (M) controls by PCR to determine the chromosomal map location of *Csrp2*. The mapping results of this analysis were deposited under accession no. MGI: 1202907 at the Jackson Laboratory and the murine *Csrp2 *gene localized to chromosome 10. In the figure, the entire T31 RH Chr 10 framework map is depicted on the left of the figure with the overall length calculated from the framework data [42]. The centromere is depicted by a black circle at the top of the map. The enlarged segment of distal Chromosome 10 is shown with respective framework markers listed to the left of the chromosome bar and a selection of mapped genes to the right. The distances between loci are calculated based on only the listed data sets, and unscored radiation hybrid cell line data are inferred where the data on either side of the missing score are in agreement. Blocks of human synteny are indicated to the right of the RH map, based on information from the NCBI's locus link [43]. Note all locus names should be in italics, but are shown in plain text for readability.

By use of the T31 mouse/hamster radiation hybrid (RH) panel [21,22] containing a set of 100 different DNAs from somatic cell hybrids, we localized the murine *Csrp2 *gene to Chromosome 10. In this analysis with the highest anchor LOD of 23.2 was assigned to D10Mit150 with a best-fit location between markers D10Mit203 proximal and D10Mit150 central, confirming the gene position that was recently launched by the Mouse Genome Sequencing Consortium (NT_09500). Noteworthy, this region is syntenic to human chromosomal region 12q21.1 (Fig. 1), essentially the region to which the human orthologue was previously assigned [23].

### Targeted disruption of the *Csrp2 *gene

**Figure 2:**
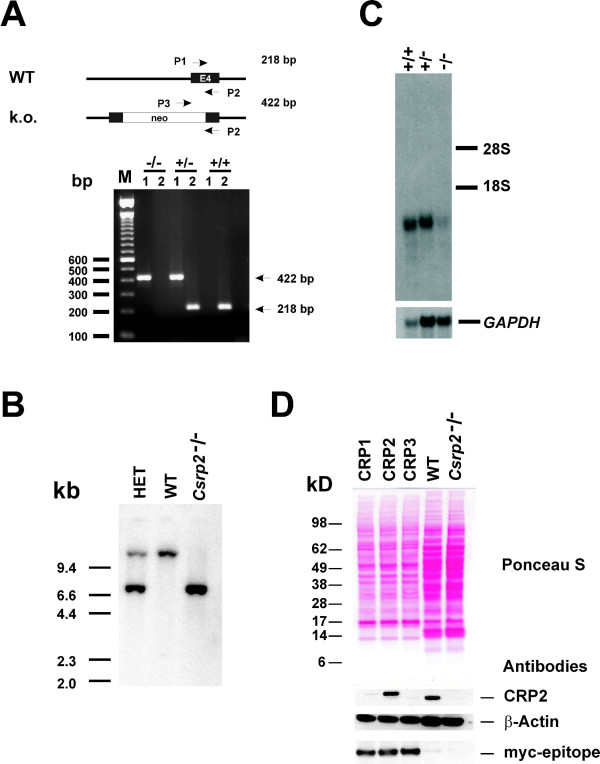
**Targeted disruption of the mouse *Csrp2 *gene**. **(A) **A neomycin resistance cassette (neo) was incorporated into *Csrp2 *(exon 4) as outlined in the Method section. The different *Csrp2 *alleles (WT *vs *k.o.) from progeny of mating heterozygotes were discriminated by PCR analysis of tail biopsy DNAs. The positions of the oligonucleotide primers used for amplification of the wild-type (218 bp) and mutant (422 bp) PCR fragments are indicated. In the genotyping experiment shown, the different DNAs were genotyped as homozygous null (-/-), heterozygous (+/-) or wild-type (+/+). **(B) **Southern hybridisation of littermate offspring from heterozygous intercrosses genotyped as heterozygote (WT), wild-type (WT), or homozygote null (*Csrp2*^-/-^). The DNA was digested with *Bam*HI, fragments were separated in a 1% agarose gel and transferred to a nylon membrane. The blot was hybridized with an external 1.5 kbp *Xho*I probe (see Methods), resulting in fragments of ~7.3 (knock out) or ~12.6 kb (wild-type) in size. **(C) **Northern blot of total kidney RNA isolated from wild-type (+/+), heterozygote (+/-), and homozygote (-/-) *Csrp2 *null mice. The RNAs were hybridized with a *Csrp2 *specific cDNA probe. The autoradiograph showed the typical 1.2. kb *Csrp2 *signal in *Csrp*2^+/+^, a weaker band with *Csrp2*^+/-^, and a faint band with the *Csrp2*^-/- ^mice. To verify the integrity of RNAs, the blot was subsequently hybridized with a *GAPDH*-specific cDNA probe. **(D) **Western blot of kidney homogenates extracted from wild-type (WT) and mutant mice (*Csrp2*^-/-^). As a positive control and to demonstrate the specificity of the CRP2 specific antibody, cell extracts taken from COS-7 cells that were transfected with myc-epitope tagged version of murine CRP1, CRP2 and CRP3 were taken. The expression of these proteins was demonstrated by subsequent probing with a myc-epitope specific antibody. In the *Csrp2 *nulls, no CRP2 band at any size was detected.

For generation of the targeting vector we isolated and sequenced a ~17.3 kbp genomic clone of the murine *Csrp2 *gene [Genbank:AY533303]. The targeting construct was generated by insertion of a neomycin resistance cassette into the *Stu*I restriction site of exon 4 and contained 1386 bp upstream and 14413 bp downstream sequences (for more details see Method section). After transfection of ES cells with the targeting construct, we tested 142 transformants by Southern blot using an external hybridization probe. Sixteen of these ES clones were found to have incorporated the targeted vector by homologous recombination. Subsequently, ES clones carrying the disrupted *Csrp2 *gene were injected into C57BL/6J blastocysts, and transferred into the uteri of pseudopregnant recipients. The mouse chimeras were identified by the inspection of the proportion of coat agouti skin color ranging from complete white to near 80% black. Crosses between chimeras and C57BL/6J mice revealed that the ES cell genome was transmitted through the germline, as indicated by the agouti skin color of the offspring. F1 hybrids tested to be heterozygous for the disrupted gene were backcrossed (up to N10) into the C57BL/6J strain background. To generate *Csrp2 *null mice, we interbred heterozygous animals and genotyped litters after weaning at 4 weeks of age using a genotyping PCR strategy (Fig. 2A). Furthermore, we performed Southern blot hybridization using an external probe to demonstrate that the targeting construct was correctly inserted into the *Csrp2 *locus (Fig. 2B). To demonstrate the absence of specific *Csrp2 *transcripts, we performed Northern blot analysis (Fig. 2C) and quantitative PCR (Additional file 1) revealing that the level of *Csrp2 *mRNA was half that of the wild type in heterozygous *Csrp2*^+/- ^null mouse. However, an aberrant RNA species that appeared at very low level was expressed in gene-disrupted mice (cf. Fig. 2C). Sequence analysis of a cDNA generated by reverse transcription of the respective mRNA species revealed that this aberrant message was generated by artificial splicing of exon 3 to the downstream neomycin/exon4 boundary (Additional file 1C). This RNA does not produce any protein product at any size as tested by Western blot analysis (Fig. 2D).

### CRP2 deficient mice are viable and fertile

Mice homologous for the targeted deletion had no gross phenotypic abnormalities, and development and reproductive function appeared normal. DNA analysis of 489 progeny (225 females, 264 males) derived from *Csrp2*^+/- ^intercrosses revealed that *Csrp2*^-/- ^mice were born in the predicted 1:2:1 Mendelian distribution (128 wild type, 243 heterozygotes, and 118 nulls). Mating of homozygous males with homozygous females produced viable offspring of normal litter size at normal frequency indicating that CRP2-deficient mice were fertile and pregnancies were carried out to full term. To detect possible structural defects induced by the absence of *Csrp2*, we comparatively examined formalin-fixed tissue sections of adult wt and *Csrp2*^-/- ^mice. We found that sections taken from kidney, skeletal muscle, heart, liver, lung, brain, thymus, stomach, or intestine were indistinguishable from those of control mice (Additional file 2, and not shown).

### Detailed analysis of heart architecture

**Figure 3:**
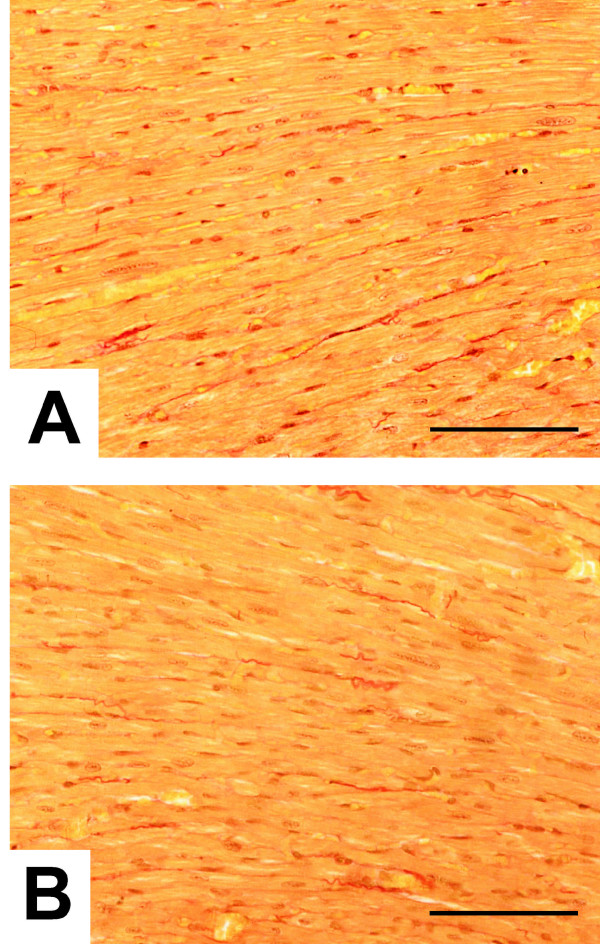
**Alterations in cardiomyocyte thickness and intercalated disc morphology in *Csrp2*^-/- ^mice**. Morphometric analysis of longitudinal cut areas of the left ventricular wall below the aortic valve from wild-type **(A) **and *Csrp2*^-/- ^**(B) **siblings were analysed for cardiomyocyte diameters using a microscope with an internal size scale. For details refer Material and Method section and Table 1. The space bar in each figure part represents 100 μM.

Previous reports have demonstrated that the absence of another CRP family member (i.e. CRP3/MLP) reproduces the morphological and clinical picture of dilated cardiomyopathy and heart failure in humans [7]. Furthermore, independent studies have shown that *Csrp2 *expression is detectable in both vascular and venous smooth muscle cells and in cardiomyocytes throughout embryogenesis suggesting an important role for *Csrp2 *in the developing heart and cardiovascular system [18,19]. Therefore, we decided to direct our attention to potential alterations of the heart. Compared to *Csrp3/Mlp*^-/-^, the *Csrp2 *nulls had no apparent degeneration or enlargements, and the weight and size of hearts taken from *Csrp2*^-/- ^mice were indistinguishable from control littermates. However, a more detailed morphometric analysis of myocardial sections revealed that the thickness/diameter of (longitudinally cut) cardiomyocytes was significantly higher in the *Csrp2*-disrupted mice (wt: 15.5 ± 0.8; 15.8 ± 1.0; 15.8 ± 1.1; 15.2 ± 1.1 *versus *ko: 17.4 ± 1.1; 17.5 ± 1.3; 17.0 ± 0.9 μm) (Fig. 3, Table 1).

**Table 1:** Morphometric analysis of myocardial sections

No. of HPF	thickness/diameter (μm) of cardiomyocytes						
	
	WT	WT	WT	WT	Csrp2^-/-^	Csrp2^-/-^	Csrp2^-/-^
1	14.6	16.7	15.9	15.9	16.7	16.3	17.1
2	16.7	16.3	16.3	14.2	20	19.4	17.5
3	14.9	13.7	17.1	14.2	17.9	20	16.3
4	15.2	15.9	14.2	14.6	19.4	17.9	16.3
5	14	14.2	14.2	14.9	15.6	15.6	15.9
6	16.7	15.6	15.9	13.5	15.9	16.7	18.4
7	16.7	15.2	15.2	13.7	19.4	17.5	17.1
8	15.2	15.9	14.2	14.2	17.1	17.5	18.9
9	14.9	16.7	17.5	17.5	18.9	16.7	16.7
10	14.2	17.5	17.5	16.7	17.1	19.4	17.9
11	15.9	16.7	15.9	15.9	17.5	20	17.9
12	15.6	15.9	15.6	15.6	17.9	17.9	17.5
13	14.9	16.3	15.2	14.9	15.6	17.9	17.1
14	14.9	17.5	15.9	15.2	15.9	15.6	17.9
15	16.7	16.7	14.9	14.2	16.7	16.3	17.1
16	14.9	14.9	15.9	14.6	18.9	17.1	16.7
17	16.3	15.6	15.6	14.6	17.9	17.9	15.6
18	15.9	14.9	14.2	17.1	15.9	16.7	15.2
19	15.2	15.2	17.1	16.7	17.1	16.7	16.3
20	15.6	14.2	17.5	15.9	15.9	16.3	15.9
Mean	15.5	15.8	15.8	15.2	17.4	17.5	17.0
SD	0.8	1.0	1.1	1.1	1.1	1.3	0.9

Abbreviation used: HPF, high-power field.

**Figure 4:**
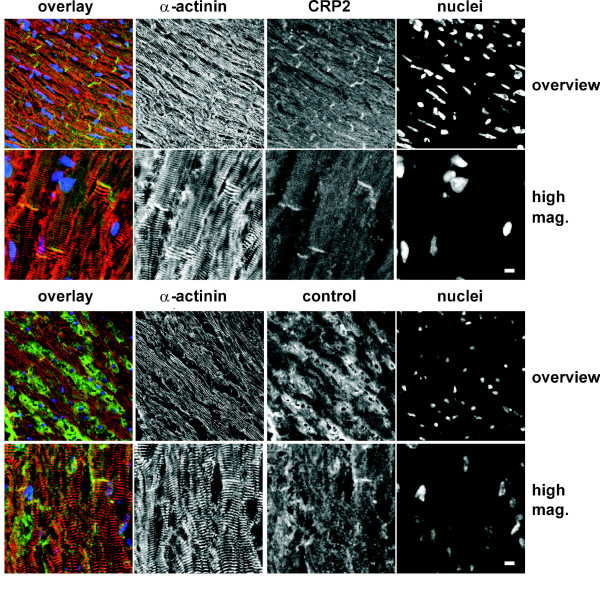
**CRP2 is preferentially associated with the intercalated disc**. Confocal micrographs of immunostained sections from wild type mice show that a strong signal for CRP can be detected at the intercalated disc (green signal in overlay in top two rows). Preimmune serum only picks up extracellular matrix in control sections (green signal in bottom two rows). The sections were counterstained for the Z-disc protein α-actinin (red signal in overlays) and with DAPI to visualise the nuclei (blue signal in overlays). The space bar represents 10 μM.

**Figure 5:**
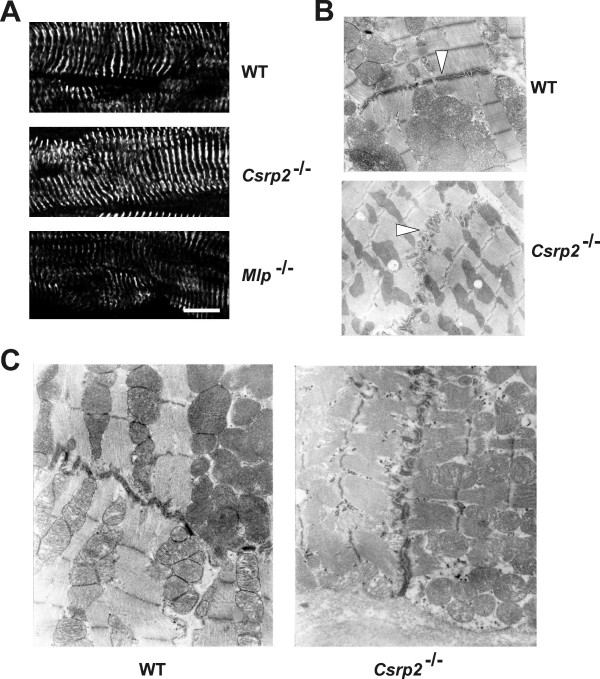
**Structure of the Z discs from wild-type, *Csrp*2^-/-^, and *Csrp3/Mlp*^-/- ^mice**. **(A) **The structures of Z discs from wild-type, *Csrp*2^-/-^, and *Csrp3/Mlp*^-/- ^were displayed by immunofluorescence microscopy using an antibody directed against sarcomeric α-actinin. The space bar represents 10 μM.**(B) **Heart sections of wild-type mice (WT) and *Csrp2 *nulls (*Csrp2*^-/-^) were infiltrated with an expoxy resin and examined in a Philips TEM 400 transmission microscope. The intercalated discs are each marked by arrowheads. Note the moderate and pronounced convolution of the membrane at the intercalated disc of *Csrp2 *null compared to wild type mice. (Original magnification × 9.000). **(C) **Heart sections of wild-type mice (WT) and *Csrp2 *nulls (*Csrp2*^-/-^) at higher magnification (× 18.000). For electron microscopic analysis three hearts taken from each genotype were analysed. The most representative images are shown in **(B) **and **(C)**.

**Figure 6:**
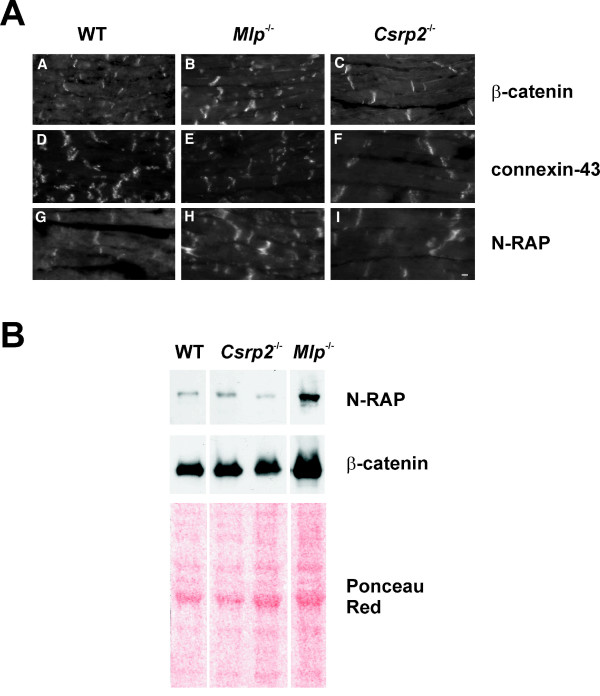
**Markers of heart dysfunction**. **(A) ***Csrp2 *nulls show less pronounced alterations in the distribution of intercalated disc-associated proteins than observed in *Csrp3/Mlp *disrupted mice. Micrographs of longitudinal frozen sections of ventricular tissues from wild-type (panel *A, D, G*), *Csrp3/Mlp*^-/- ^(panel *B, E, H*) and *Csrp2*^-/- ^(panel *C, F, I*) mice, stained with antibodies against β-catenin (panel *A-C*), connexin-43 (panel *D-F*) and N-RAP (panel *G-I*) antibodies. While β-catenin and N-RAP expression are significantly upregulated in *Csrp3/Mlp *deficient mice (panel B, H) and to a lesser extent in *Csrp2*^-/- ^mice (panel C, I), connexin-43 expression is reduced in *Csrp3/Mlp*^-/- ^as well as *Csrp2*^-/- ^mice (panel F). The space bar represents 10 μM.**(B) **Western blot analysis of N-RAP and β-catenin. Heart extracts from normal (WT), *Csrp2*- and MLP-deficient mice were probed with antibodies specific for N-RAP and β-catenin. Equal loading was demonstrated in Ponceau Red stain.

Based on the knowledge that the Z discs of *Csrp3/MLP*^-/- ^mice show misalignment and the fact that CRP2 has affinity for α-actinin [15,6], we next envisaged to comparatively analyse the Z-disc defining the lateral boundaries of the sarcomere. To do so, we first tested if CRP2 is expressed in adult cultured cardiomyocytes (Additional file 3). This analysis revealed that, compared to the regular arrangement of α-actinin and F-actin, CRP2 showed a more irregular staining pattern in cultured cardiomyocytes. In the *in vivo *situation, CRP2 was most prominently localized at the intercalated disc (Fig. 4). However, in contrast to *Mlp*^-/- ^cardiomyocytes in which the myofibrils are somewhat disorganized, the localization pattern of α-actinin, an integral component of the Z-disc is indistinguishable in wild-type and *Csrp2*^-/- ^cardiomyocytes (Fig. 5A). Furthermore, the intercalated discs were more convoluted in *Csrp2*^-/- ^mouse hearts than in hearts of wild type littermates (Fig. 5B) but less pronounced than in the *Csrp3/Mlp *null mice (not shown). We next addressed the question whether a lack of CRP2 expression would lead to a change of the molecular composition of the intercalated disc in addition to its altered ultrastructure, as previously described for the *Csrp3/Mlp *knock out mice [7,24]. Although less pronounced than in the *Csrp3/Mlp *knock out mice, we found that *Csrp2 *knock outs show also increased signal for the adherens junction protein β-catenin at the intercalated disc, while there is less signal for connexin-43, the component of the gap junctions (Fig. 6A). Likewise, the slight increase of cardiomyocyte thickness in *Csrp2*^-/- ^mice were detectable confirming the diameter measurements described above. To test if the increased signals determined for N-RAP and β-catenin are due to a higher expression of these proteins or an altered distribution, we performed Western blot analysis (Fig. 6B). We found that the expression of both proteins was unmodified compared to normal control mice, while we confirmed the increase in expression of both proteins in *Csrp3*^-/-^/*Mlp*^-/- ^deficient mice.

### Expression of *Csrp1 *and *Csrp3 *in CRP2 deficient mice

To investigate whether expression of *Csrp1 *and *Csrp3 *was affected by the absence of CRP2, we isolated RNAs from different organs and compared the transcript levels of respective genes in wild-type and homozygous mutant mice. Total RNA was isolated from different tissues and the relative amounts of *Csrp1 *and *Csrp3/Mlp *mRNAs were determined by Northern blot analysis. We found that the transcriptional activity of *Csrp1 *and *Csrp3 *genes in *Csrp2*^-/- ^mice was indistinguishable from controls (Additional file 4).

## Discussion

CRP2 belongs to the CRP family of LIM domain proteins that are evolutionarily conserved. The sequence of mouse CRP2 displays 99.5%, 97.9%, and 97.4% amino acid identity to human, quail, and chicken CRP2, respectively. Therefore, it is supposed that this LIM domain protein has a critically, evolutionarily conserved role for the development or maintenance of vital processes within organisms. Consistent with this hypothesis is the observation that *Csrp2 *expression is detectable in a number of cell types during embryogenesis, including mesenchyme, vascular smooth muscle cells, and epithelial derivatives [19]. It was also demonstrated that *Csrp2 *is expressed in the cardiovascular system at early time points during mouse development indicating an important role in vascular smooth muscle differentiation [18]. Based on the knowledge that CRP2 and other CRPs can bind to zyxin, α-actinin, and α-actin [25,6,26], it is tempting to speculate that these proteins are bridging molecules that are important for the organization or stabilization of cytoskeletal structures. This is also supported by structural data showing that the two tandemly arranged LIM domains are completely independent folded units that are linked by a highly flexible spacer [11,12].

Moreover, *Csrp2 *was originally identified from normal quail embryo fibroblasts that were screened for genes suppressed in the course of oncogenic transformation [3]. Subsequently, it was demonstrated that the transcriptional suppression of *Csrp2 *is generally linked to the transformed state of cells [27] suggesting that CRP2 might have tumor-suppressor activity. In this context it is remarkable that the reintroduction of CRP2 into human breast and colorectal cancer cell lines was sufficient to significantly decrease colony formation [28]. However, all these findings demonstrate that indeed CRP2 must have specific functions both in development and control of cancer cell growth. Considering the postulated roles and the observed expression pattern of CRP2, the finding that mice lacking a functional *Csrp2 *gene exhibit a quite mild phenotype was unexpected but is in agreement with a recent report characterizing a similar *Csrp2 *gene disruption in mice [20]. No gross morphological or physiological abnormalities were detected, suggesting that *Csrp2 *is dispensable for normal development. This finding is somewhat surprising, since the ablation of the CRP3/MLP or TLP genes is associated with striking morphological and functional alterations [7,5]. Possibly, there exists a functional redundancy that is known from other myogenic factors [29,30]. This would give rise to "cryptic robustness" to cells and organs expressing different CRPs. Conversely, a loss of function mutation or an experimental inactivation of a *Csrp *gene would predominantly affect cells or organs exclusively expressing this family member. There are some good arguments supporting this "quantitative model of CRP function". First, different independent studies have shown that CRP3/MLP is exclusively expressed in heart at high level and to a lower extent in skeletal muscle [4,18,8,31]. The abundance of *Csrp3/Mlp *transcripts in these organs is several times higher than those of *Csrp1 *and *Csrp2 *(Additional file 4). Therefore, it is reasonable that the CRP quantities expressed by these genes cannot compensate for CRP3/MLP. Consistent with our hypothesis, it is not surprising that *Csrp3/Mlp *nulls have a strong cardiac phenotype [7] and *Csrp3/Mlp *mutations are associated with heart failure in humans [14,15]. Secondly, TLP is highly expressed in the thymus and accordingly mice deficient for TLP have alterations of normal thymus function [5]. Third, our data presented in this study indicate that the loss of CRP2 has only a mild cardiac phenotype. Compared to the *Csrp3/Mlp *knock out model [7], the cardiac alterations are much less pronounced. The localisation of CRP2 (Fig. 4) and the alterations in tissue distribution of N-RAP and β-catenin found in animals lacking CRP2 (Fig. 6) point to a functionality of CRP2 in the organisation of the intercalated disc. In this regard, CRP2 might cooperate with other CRPs. It is tempting to speculate that during heart development *Csrp3/Mlp *or *Csrp1 *may be in the position to compensate for the loss of CRP2. This principle may also be true for the development of other organs. *Csrp2 *is broadly expressed in mouse embryos and adults [18,19,31]. Likewise, *Csrp1 *is widely expressed in mouse tissues [18,32] and expression coincides with that of *Csrp2 *(Additional file 5). Noteworthy, the *Csrp1 *and *Csrp2 *genes are expressed in early vertebrate embryos and are spatially regulated in mouse [19,32]. In this regard, the recent finding that both, CRP1 and CRP2, are able to convert pluripotent 10T1/2 fibroblasts into smooth muscle cells [9] demonstrates that these CRPs share some functions. Together, these observations are in agreement with the notion that individual CRPs share redundant functions and may explain the mild phenotype of the *Csrp2*^-/- ^mouse and the surprising observation that *Csrp2 *thought to be involved in key biological processes could be removed without affecting the organism.

A recent report has shown that CRP2 physically associates with other cardiovascular lineage regulators, such as serum response factor (SRF) and GATA proteins, thereby synergistically activating the transcription of smooth muscle cell target genes (i.e. smooth muscle myosin heavy-chain, calponin, smooth muscle α-actin, caldesmon, SM22α) with contractile abilities [17]. Therefore, it is possible that the lack of CRP2 cause alterations in expression of respective genes resulting in cardiomyocytes displaying a slight increase in thickness. CRP2 and its close homologue CRP1 are co-expressed during early cardiovascular development and both CRPs share similar functionality in facilitating transcriptional activity of the SRF-GATA-complex, especially of promoters encoding the SMC target genes [9]. Therefore, it is reasonable that CRP1 in the *Csrp2 *nulls can substitute for CRP2 resulting in the observed mild phenotype. Noteworthy, this functional substitution is not overall complete. In preliminary echocardiography using five animals each we found indications that the disruption of the *Csrp2 *gene is associated with a lower left ventricular wall thickness and fractional shortening (Additional file 6) indicating reduced heart function in respective nulls.

However, the lack of an "obvious *Csrp2*^-/- ^phenotype" in an animal kept under laboratory conditions constitutes no compelling argument against a unique and indispensable role of a gene in the normal physiology and the specialized *in vivo *function of CRP2 may become apparent only after appropriate challenges to the mice. In this context, it might be significant that apart from the shared binding affinity of CRP1, CRP2 and CRP3/MLP to zyxin and α-actinin, we and others have isolated proteins specifically interacting with individual CRPs [33,8,34,10]. The ability of CRPs to discriminate between different target proteins may the basis for subtle differences in functionality. Such an overlap of functional redundancy with protein specific activities was also demonstrated in the myogenic basic helix-loop-helix (HLH) genes [29,30]. Comparable to the *Csrp2*^-/- ^nulls, the inactivation of *MyoD *leads to up-regulation of the myogenic HLH gene *Myf-5 *and results in normal development [29]. It is possible that during early development and differentiation of different myogenic lineages, myogenic factors and also CRPs act cooperatively, but exert distinct function at later stages, when the fine-tuning of cellular programs happens.

The demonstration that the *Csrp2 *gene is silenced during cellular transformation and CRP2 protein induces growth-inhibitory effects when overexpressed in cancer cells points to a critical role in the control of normal cell growth. Future studies will clarify whether mice or cells lacking CRP2 will be more susceptible to tumor promoters or other physiological stress factors. Moreover, the phenotype of mice lacking several members of the CRP family will be highly informative as it directly addresses the question of mutual functional substitution and redundancy.

## Conclusion

We conclude that the LIM domain protein CRP2 is involved in normal cardiomyocyte development. The loss of CRP2 influences the fine architecture of the intercalated disc morphology.

## Methods

### Isolation of murine *Csrp2 *sequences

By using a cDNA specific for rat *Csrp2 *as a probe, we isolated different genomic *Csrp2 *clones from a mouse genomic 129SvJ library. The overall organization of the *Csrp2 *gene was in agreement with previous reports [35]. The sequence of a ~17.3 kbp fragment of one clone (clone 5o) containing exon 3 through 6 of *Csrp2 *was sequenced and deposited [GenBank: AY533303].

### Chromosomal Assignment of murine *Csrp2 *using the T31 radiation hybrid mapping panel

DNAs from one hundred cell lines of the T31 mouse radiation hybrid (RH) panel (Research Genetics, Invitrogen, Paisley, UK) were typed using two independent sets of A23 hamster and 129 mouse DNA controls. The individual genomic PCRs were performed in reactions (50 μl) containing 50 ng of DNA, 50 mM KCl, 10 mM Tris-HCl, pH 8.3, 1.5 mM MgCl_2_, 400 μM of each dNTP, and 2 Units *Taq *polymerase (Roche), respectively. Primers used were: 5'-d(GAGACCGACATCTTAGGACAG)-3' and 5'-d(GATTGTGATGAGCTGCAGGCG)-3'. PCR conditions were: initial denaturation at 95°C for 5 min, 35 cycles of amplification (94°C for 1 min, 50°C for 1 min, 72°C for 3 min), final extension at 72°C for 10 min, and cooling to 4°C. The PCR products were separated on 1.8% agarose in 1× TBE and visualized by ethidium bromide staining. Data from this whole-genome radiation hybrid mapping were electronically submitted, analyzed, and deposited [MGI: 1202907] at the Jackson Laboratory, Bar Harbor, MA [36].

### Gene targeting

The targeting construct was created by a two-step cloning strategy (see also Additional file 5). In a first step, the 3.4 kbp *Bgl*II fragment containing nt 4984 to nt 8393 [Genbank: AY533303] was subcloned and a blunted 1.7-kb *Xho*I/*Sal*I fragment containing a neomycin resistance cassette was cloned into the *Stu*I-site at nt 7292. Subsequently, the enlarged fragment was cloned back into the original 17.3 kbp genomic clone. All cloning boundaries were verified by sequencing. The resulting targeting vector was digested with *Xho*I cutting at position 1506 and in vector pBS-SKII. The 1.5 kbp *Xho*I fragment was removed by gel electrophoresis and later applied in Southern blot analysis as 5' external probe, and the remaining targeting vector was transfected into embryonic stem (ES) cells (129 SvJ) by electroporation. ES cells that had incorporated the transgene were subsequently maintained on mitotically inactive mouse embryonic fibroblast feeder layers and selected in the presence of 400 μg G418/ml. A total of 142 neomycin-resistant ES clones were picked, and their genomic DNA was isolated, digested with *Bam*HI, separated on 1.0% agarose gels, and transferred to Hybond-N membranes (Amersham Pharmacia, Braunschweig, Germany). For the identification of homologous recombinants, Southern blots were performed using the ^32^P-labeled ~1.5-kbp external 5' fragment as a probe. The ES cell clones that showed correct targeting were injected into C57BL/6J blastocysts. Subsequently, the composites were transferred into pseudopregnant foster mice and resulting chimeras were mated. The congenic strain was produced by repeated backcrosses into the C57BL/6J strain and embryos and sperms from N10 generation were cryoconserved [EM: 01784] by the European Mouse Mutant Archive [37].

### Genotyping

Genomic DNA isolated from tail biopsies of the offspring were genotyped by PCR using primer combinations 5'-d(CAGCAGTAGAGCTCCGAAGCTCC)-3' (ex4for) and 5'-d(CTACCTTCCCAGCTCCAATGATC)-3' (ex4rev), or primer combination 5'-d(CTGCTCTTTACTGAAGGCTCTTT)-3' (neofor) and ex4rev resulting in fragments of 218 (wild-type) or 422 bp (k.o.), respectively.

### Quantitative analysis of *Csrp2 *transcripts

Gene expression of *Csrp2 *was monitored by real-time PCR as described in detail elsewhere [31]. To correct for differences in quantity between RNA samples, data of amounts of *Csrp2 *transcripts were normalized to those of β-actin.

### Isolation and immunostaining of cardiomyocytes

Primary cultures of cardiomyocytes were prepared, maintained and stained as described previously [38]. The mouse monoclonal antibodies sarcomeric α-actinin (clone EA53) and DAPI to stain the nuclei were obtained from Sigma; Alexa633-conjugated phalloidin to visualise F-actin was purchased from Invitrogen. Cy3-conjugated anti mouse and Cy2-conjugated anti rabbit antibodies were from Jacksom Immunochemicals (via Stratech Scientific, Newmarket, UK). Confocal micrographs of stained cardiomyocytes were taken in a Zeiss LSM 510 confocal microscope equipped with argon, helium-neon and blue diode lasers, using a 25×/0.8 oil immersion and a 63×/1.4 oil immersion lens, respectively.

### Histological analysis

Tissue sections from various organs were fixed with 4% paraformaldehyde in phosphate-buffered saline for 24 hours and analyzed according to standard procedures. For the analysis of murine hearts, 4 μm thick sections were cut along the frontal axis and stained with hematoxylin/eosin and Sirius red. Longitudinal cut areas of the left vetricular wall below the aortic valve were analysed for cardiomyocyte diameters using an internal microscopic size standard (mouse erythrocyte, 7 μm) and counting 20 high-power fields (HPF) of three (*Csrp*2^-/-^) or four (WT) animals. In all experiments, homozygous mutant mice were compared to wild-type siblings. Cryosections (10 μm thickness) were prepared from equivalent ventricular regions of age-matched wild-type, *Csrp2*^-/-^, and *MLP*^-/- ^mice and were immunostained for N-RAP [24,39], connexin-43 (Chemicon Int., Temecula, CA), β-catenin (Sigma-Aldrich, Taufkirchen, Germany), and sarcomeric α-actinin [40] as described previously [24].

### SDS-PAGE, immunoblotting, and generation of CRP expression plasmids

Whole-cell extracts from transfected COS-7 cells or kidney lysates were prepared following standard procedures. Equal amounts of proteins (30 μg) were resolved in NuPAGE™ Bis-Tris gels (Novex, Invitrogen, Karlsruhe, Germany) and electro-blotted onto a Protran membrane (Schleicher & Schuell). Proteins were electroblotted onto nitrocellulose membranes (Schleicher & Schuell, Dassel, Germany) and unspecific binding sites were blocked in TBST [10 mM Tris/HCl; 150 mM NaCl; 0.1% (v/v) Tween 20; pH 7.6] containing 5% (w/v) nonfat milkpowder. Primary antibodies employed were directed against the myc-epitope (M5546, Sigma), β-actin (A5441, Sigma) and CRP2 [41]. They were diluted in 2.5% (w/v) nonfat milkpowder in TBST and visualized using horseradish peroxidase-conjugated anti-mouse- or anti-rabbit-IgG (Santa Cruz) and the Supersignal chemiluminescent substrate (Pierce, Bonn, Germany). Expression vectors for murine CRP1, CRP2 and CRP3 were prepared in vector pCMV-Myc (Clontech, Heidelberg, Germany). Therefore heart mRNA was reversed transcript and specific cDNA for murine *Csrp1*, *Csrp2*, and *Csrp3 *was generated using primers Csrp1-1 TCT CCC TGG ACA GAG CAG AAT G, Csrp1-2 CTC ACT CTG AGT GAA CCA AGG C, Csrp-2-1 CTC CCT CCT CCC ACT CGG AAT G, Csrp2-2 TTA CTG GTT CAC ACC ATT ACT GAG C, Csrp3-1 TTG GCC CAG AGT CTT CAC CAT G, and Csrp3-2 AGC AGG CAG CTT CAC TCC TTC, respectively. The cDNAs were cloned into pGEM-T-Easy vector (Promega, Madison, WI), sequenced and subcloned into the *Eco*RI site of expression vector pCMV-Myc. Transfection was done using the FuGene transfection reagent (Roche, Mannheim, Germany).

### Electron microscopic studies

Tissue pieces from equivalent regions of the left ventricle taken from 8–10 month old male mice (3 animals each, 4–5 slices per animal) were fixed and prepared for electron microscopy as described before [24]. The ultrathin sections were stained with uranyl acetate, air-dried and examined with a Philips transmission electron microscope TEM 400.

### Echocardiographic measurements

Five 8-month-old male animals each (wildtype, *Csrp2*^-/-^) were anaesthetized with a combination of ketamine (100 mg/kg) and xylamine (5 mg/kg) to perform echocardiographic examination using a Sonos 5500 from Philips Medical Systems equipped with a 12 MHz transducer. The thicknesses of the anterior and posterior walls of left ventricle of respective animals were measured in the 2-D directed M-mode.

## Authors' contributions

JFS was responsible for the maintenance of the *Csrp2 *null strain including backcrossing to N10, genotyping, Northern- and Western blot experimentation, and helped in sequencing the ~17.3 kbp genomic fragment. MM has performed the mouse manipulation necessary for generation the *Csrp2 *null strain. EE has performed the experimentation for heart architecture analysis and the isolation/staining of murine cardiomyocytes. SW and DS provided their technical skills in all experiments. KG performed the quantitative real time PCR experiments. RB performed the histological analysis of mouse tissues and calculated cardiomyocyte diameters. RW cloned the disruption construct, sequenced part of the *Csrp2 *gene, performed the experiments necessary for chromosomal assignments, and drafted the manuscript.

## Supplementary Material

Additional File 1 **Quantitative real-time RT-PCR**. **(A) **Kidney RNAs from *Csrp2*^+/+^, *Csrp2*^+/-^, and *Csrp2*^-/- ^littermates were reverse-transcribed and analyzed for *Csrp2 *expression using a LightCycler protocol (*left panel*). Data acquired were normalized to β-actin and relative intensities were compared to *Csrp2*-expression in *CSRP2*^+/+ ^mice (set to 100). The relative expression of *Csrp2 *obtained by real time PCR in normal and in *Csrp2 *nulls was confirmed by Northern blot (*right panel*). **(B) **The amplicon from *Csrp2*^-/- ^mice was sequenced showing that the aberrant mRNA results from an artificial splice event between exon3 and the downstream neo/exon4 boundary inserting 25 bps. Click here for file

Additional File 2 **Tissue morphology**. Tissue slices of adult *Csrp2*^-/- ^**(A-D) **and wild type control mice **(A'-D') **taken from renal cortex **(A, A') **and pelvis **(B, B')**, skeletal muscle **(C, C')**, and liver **(D, D') **were Hematoxylin-Eosin-stained and analysed by light microscopy. The space bar in each figure represents 100 μM. Click here for file

Additional File 3 **CRP2 expression in cultured adult murine cardiomyocytes**. **(A) **Cultured murine cardiomyocytes were permeabilized and stained with an antibody specific for CRP2 or a preimmuneserum (*inlet*). The cells were washed and incubated with a second antibody that was coupled with alkaline phosphatase. After extensive washing the cells were then incubated with the fast red substrate (DAKO, Hamburg, Germany) and pictures were taken in a standard light microscope. **(B-D) **Cardiomyocytes were simultaneously stained for CRP2 **(B)**, α-actinin **(C) **and F-actin **(D) **and analysed by confocal microscopy. The space bar represents 10 μM. Click here for file

Additional File 4 **Analysis of *Csrp *expression in *Csrp2 *deficient mice**. Northern blot analysis from RNAs isolated from different organs of wild-type (+/+) and *Csrp2*^-/- ^mice were analysed for expression of *Csrp1*, *Csrp2*, and *Csrp3/Mlp*. The ethidium bromide-stained gel is shown to demonstrate equal loading of RNA samples. Click here for file

Additional File 5 **Organisation and disruption of the murine *Csrp2 *gene**. **(A) **The *Csrp2 *gene contains one non-coding (E1) and 5 coding exons (E2-E6) that are marked by white or black boxes. For cloning of the disruption construct a 17.3 kbp fragment of the *Csrp2 *gene spanning E1 to E6 was isolated and a *neo *cassette was inserted into the *Stu*I site of exon 4. For details see Materials and Method section. **(B) **The localisation of the external hybridisation probe used for verification of successful insertion by Southern blot is depicted as a solid red line. This probe detects a ~12.6 kb *Bam*HI fragment in wild type (*Csrp2*) and a ~7.3 kb *Bam*HI fragment in *Csrp2 *nulls (Mut *Csrp2*). Animals heterozygous for the disruption allele show both fragments in Southern blot analysis (cf. Fig. 2B). Click here for file

Additional File 6 Echocardiography in wildtype and *Csrp2 *nulls. Click here for file
