# Supplementary figures and images for: Targeted disruption of the mouse Csrp2 gene encoding the cysteine- and glycine-rich LIM domain protein CRP2 result in subtle alteration of cardiac ultrastructure
Source: BMC Dev Biol. 2008 Aug 19;8:80. doi: 10.1186/1471-213X-8-80 (PMC2529283; doi:10.1186/1471-213X-8-80)

**A**

relative expression [%]

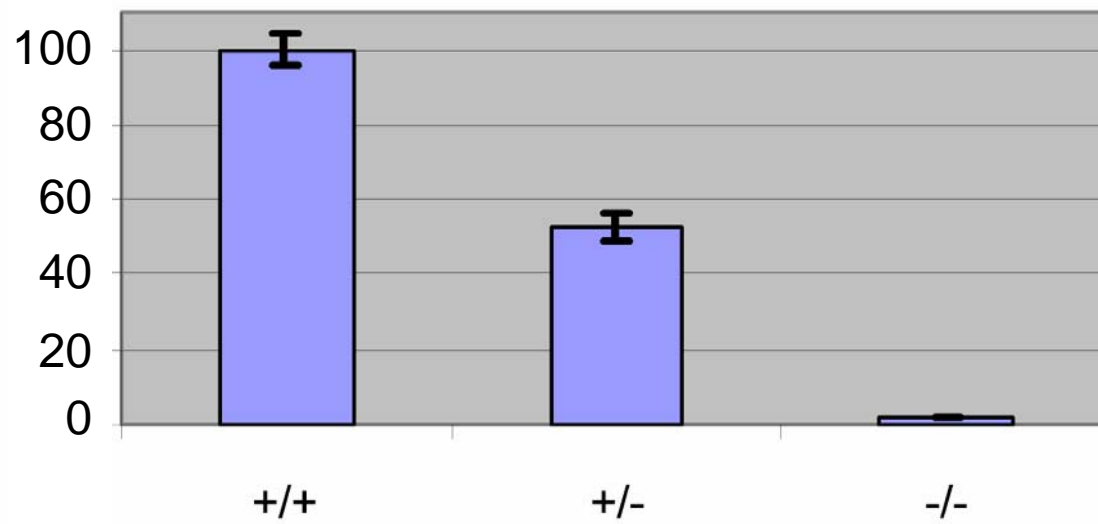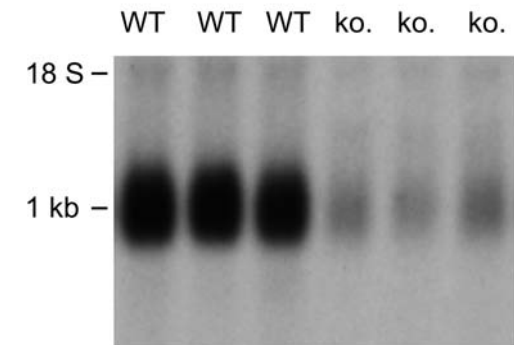**B**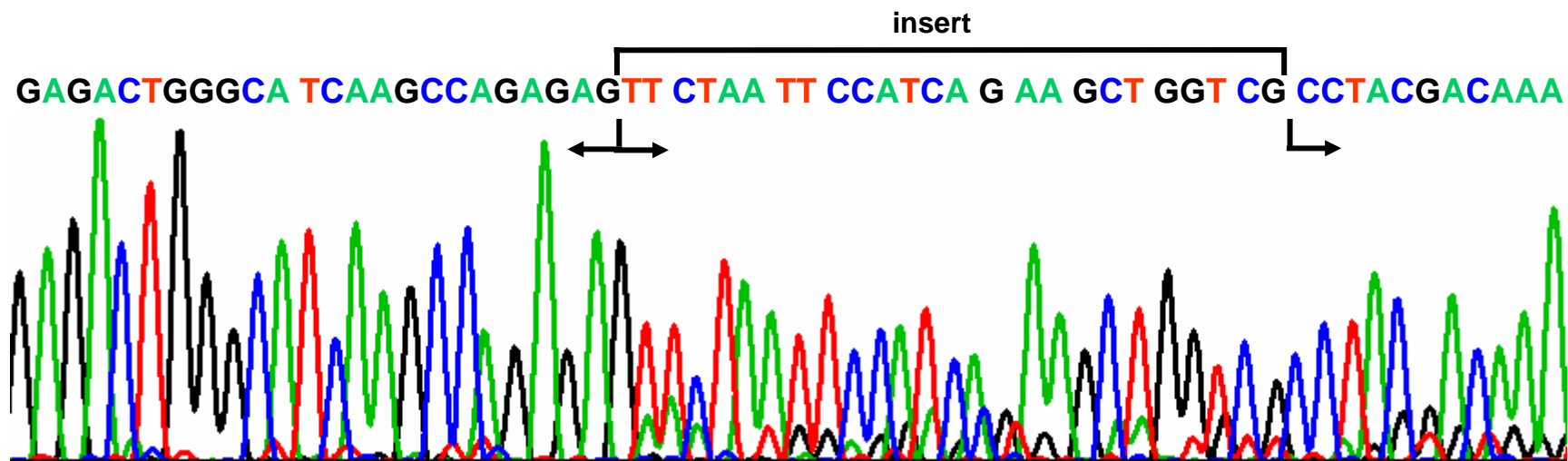

Supplement: Additional File 1 — Quantitative real-time RT-PCR. (A) Kidney RNAs from Csrp2+/+, Csrp2+/-, and Csrp2-/- littermates were reverse-transcribed and analyzed for Csrp2 expression using a LightCycler protocol (left panel). Data acquired were normalized to β-actin and relative intensities were compared to Csrp2-expression in CSRP2+/+ mice (set to 100). The relative expression of Csrp2 obtained by real time PCR in normal and in Csrp2 nulls was confirmed by Northern blot (right panel). (B) The amplicon from Csrp2-/- mice was sequenced showing that the aberrant mRNA results from an artificial splice event between exon3 and the downstream neo/exon4 boundary inserting 25 bps. [file 1471-213X-8-80-S1.pdf]

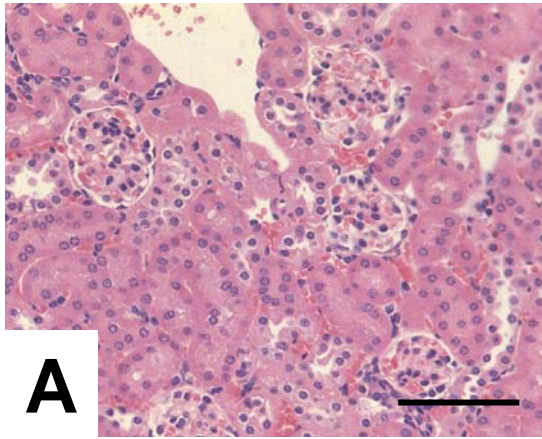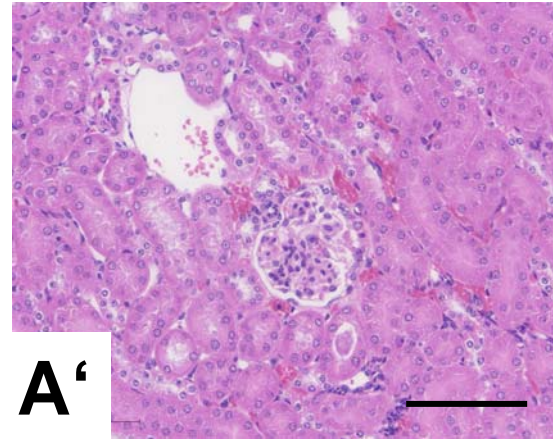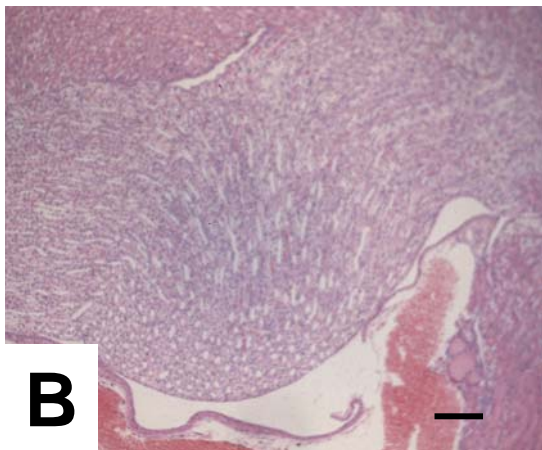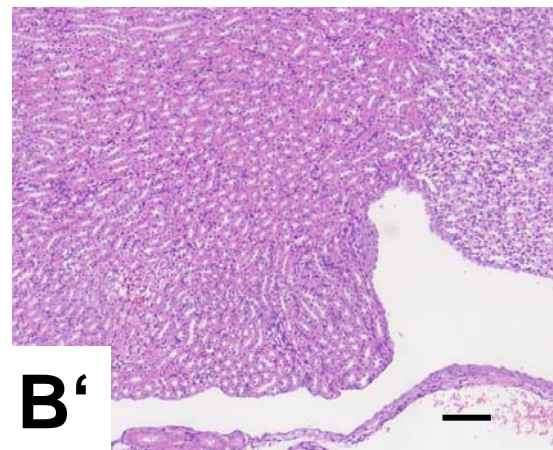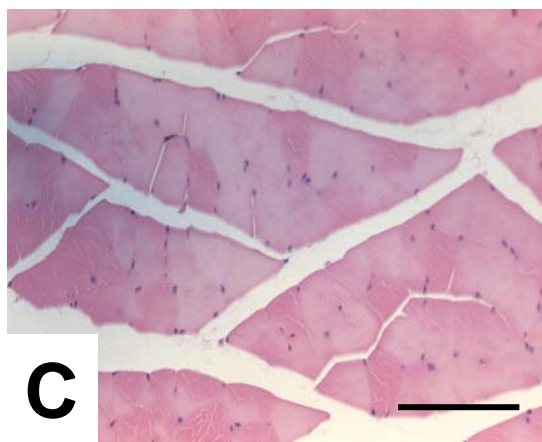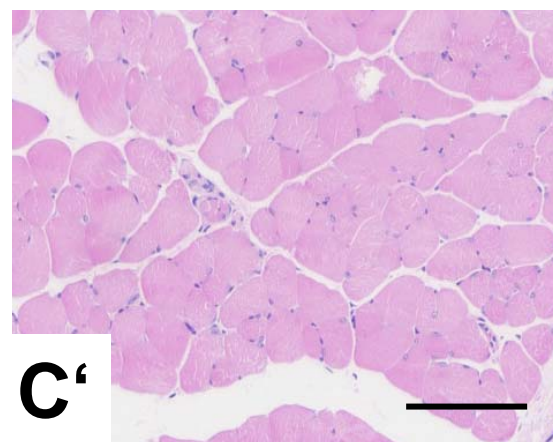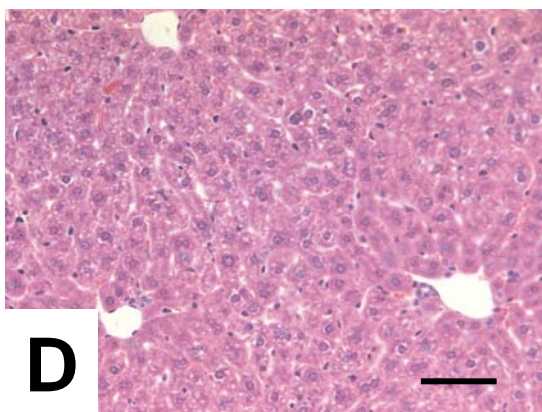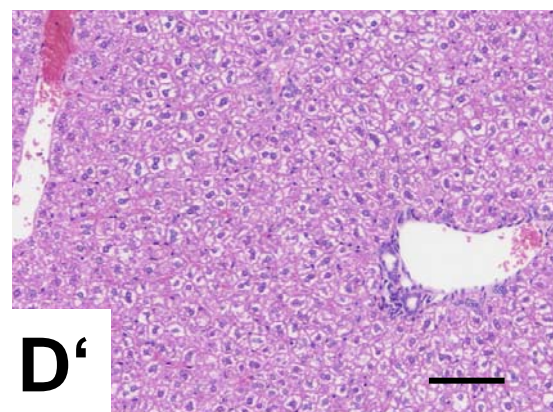

Supplement: Additional File 2 — Tissue morphology. Tissue slices of adult Csrp2-/- (A-D) and wild type control mice (A'-D') taken from renal cortex (A, A') and pelvis (B, B'), skeletal muscle (C, C'), and liver (D, D') were Hematoxylin-Eosin-stained and analysed by light microscopy. The space bar in each figure represents 100 μM. [file 1471-213X-8-80-S2.pdf]

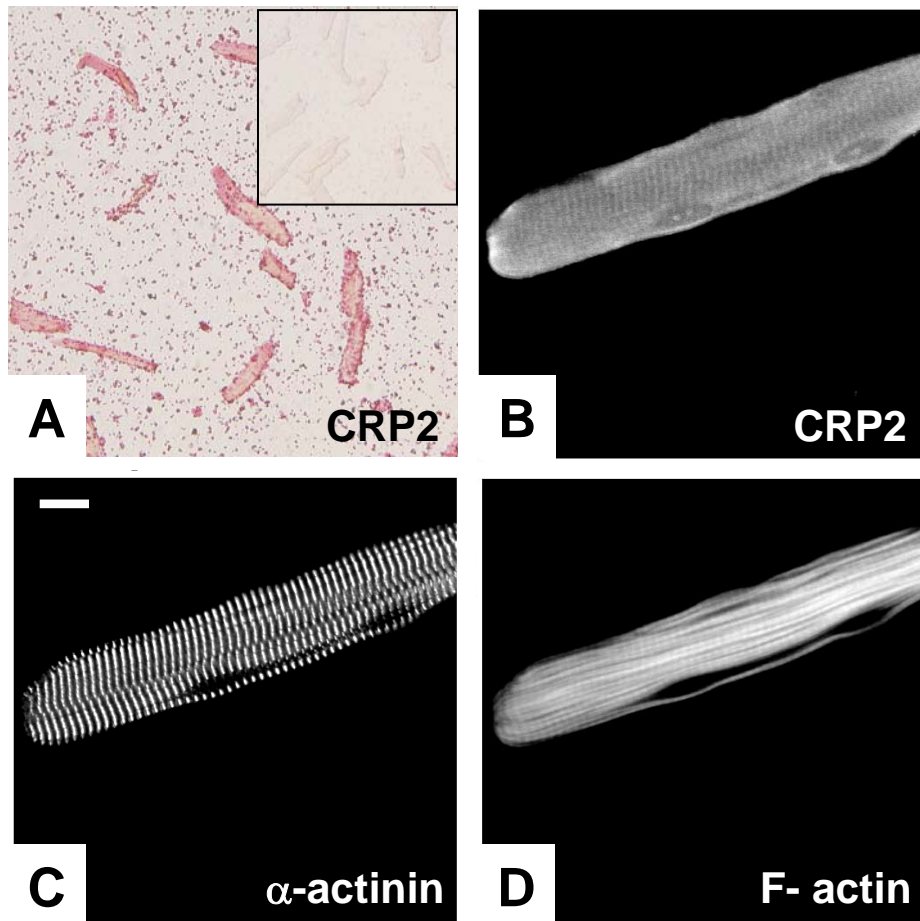

Sagave *et al.*, Supplement 3

Supplement: Additional File 3 — CRP2 expression in cultured adult murine cardiomyocytes. (A) Cultured murine cardiomyocytes were permeabilized and stained with an antibody specific for CRP2 or a preimmuneserum (inlet). The cells were washed and incubated with a second antibody that was coupled with alkaline phosphatase. After extensive washing the cells were then incubated with the fast red substrate (DAKO, Hamburg, Germany) and pictures were taken in a standard light microscope. (B-D) Cardiomyocytes were simultaneously stained for CRP2 (B), α-actinin (C) and F-actin (D) and analysed by confocal microscopy. The space bar represents 10 μM. [file 1471-213X-8-80-S3.pdf]

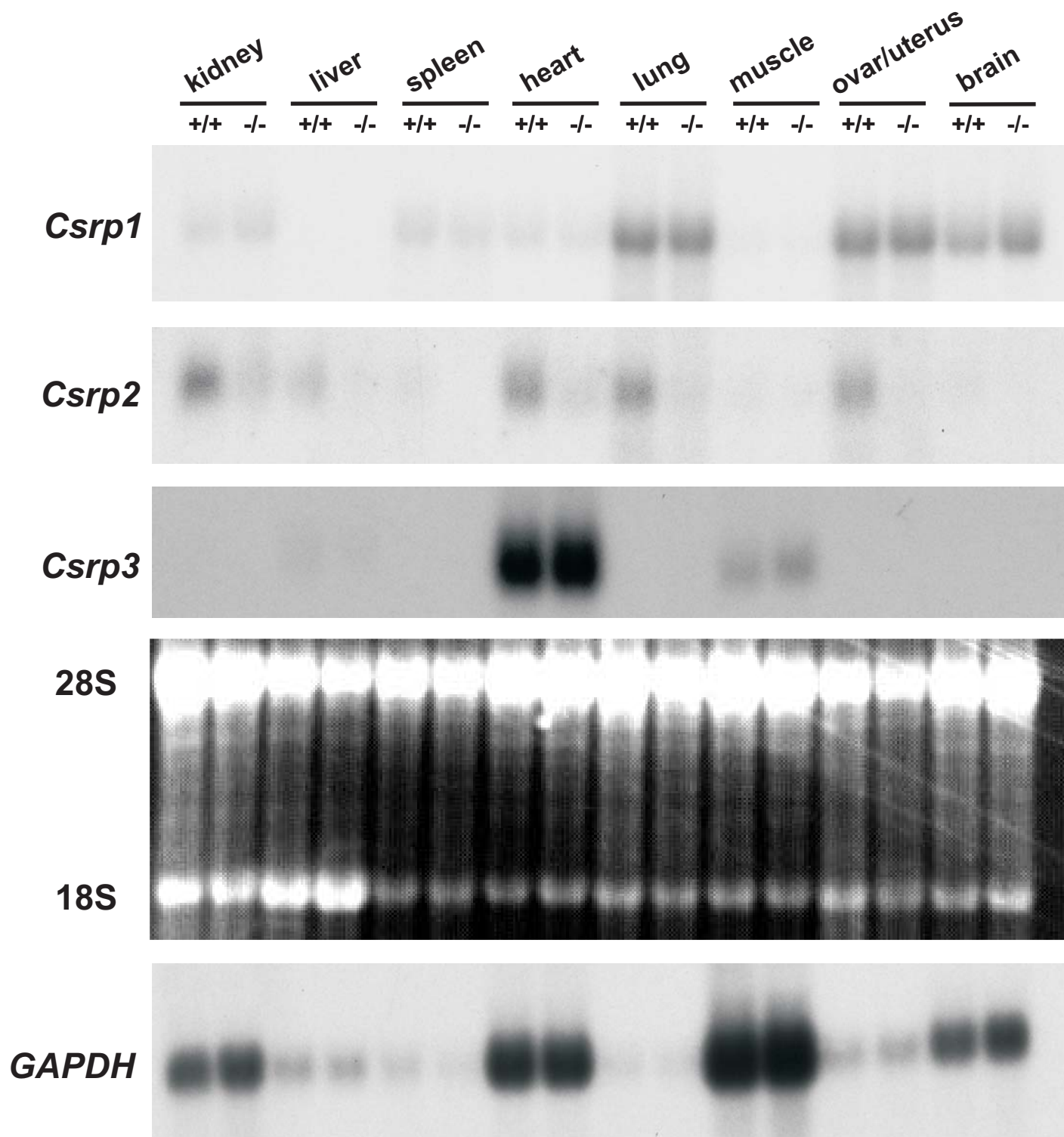

Supplement: Additional File 4 — Analysis of Csrp expression in Csrp2 deficient mice. Northern blot analysis from RNAs isolated from different organs of wild-type (+/+) and Csrp2-/- mice were analysed for expression of Csrp1, Csrp2, and Csrp3/Mlp. The ethidium bromide-stained gel is shown to demonstrate equal loading of RNA samples. [file 1471-213X-8-80-S4.pdf]

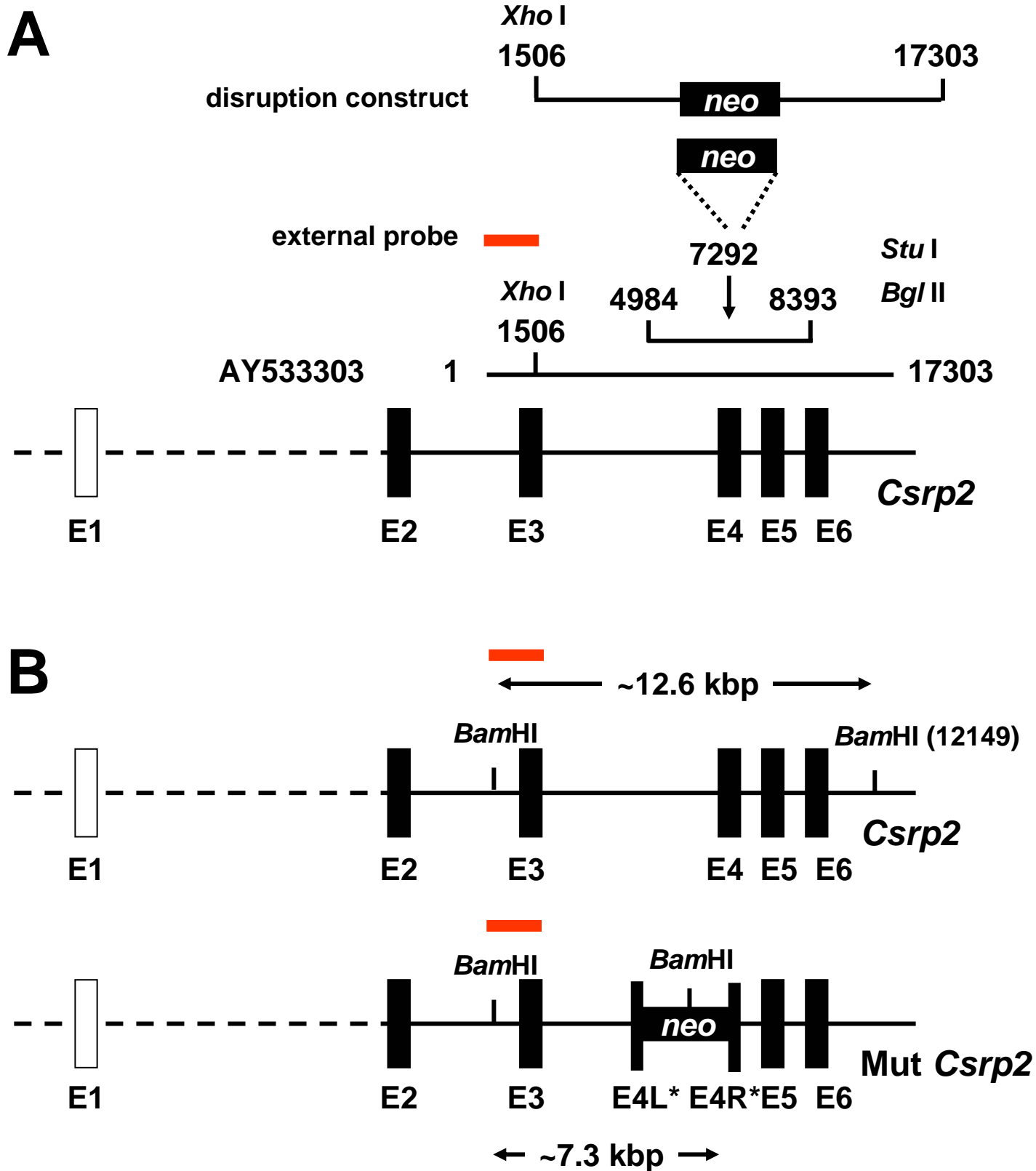

Supplement: Additional File 5 — Organisation and disruption of the murine Csrp2 gene. (A) The Csrp2 gene contains one non-coding (E1) and 5 coding exons (E2-E6) that are marked by white or black boxes. For cloning of the disruption construct a 17.3 kbp fragment of the Csrp2 gene spanning E1 to E6 was isolated and a neo cassette was inserted into the StuI site of exon 4. For details see Materials and Method section. (B) The localisation of the external hybridisation probe used for verification of successful insertion by Southern blot is depicted as a solid red line. This probe detects a ~12.6 kb BamHI fragment in wild type (Csrp2) and a ~7.3 kb BamHI fragment in Csrp2 nulls (Mut Csrp2). Animals heterozygous for the disruption allele show both fragments in Southern blot analysis (cf. Fig. 2B). [file 1471-213X-8-80-S5.pdf]
