# Supplementary material for: Targeted disruption of the mouse Csrp2 gene encoding the cysteine- and glycine-rich LIM domain protein CRP2 result in subtle alteration of cardiac ultrastructure
Source: BMC Dev Biol. 2008 Aug 19;8:80. doi: 10.1186/1471-213X-8-80 (PMC2529283; doi:10.1186/1471-213X-8-80)
Supplement: Additional File 6 — Echocardiography in wildtype and Csrp2 nulls. [file 1471-213X-8-80-S6.doc]

**Additional file 6**

**Echocardiography in wildtype and *Csrp2* nulls**

| **Animal** | **FS (%)** | **Anterior wall of**  **the left ventricle**  **(cm)** | **Posterior wall of**  **the left ventricle**  **(cm)** |
| --- | --- | --- | --- |
| WT1 | 59 | 0.146 | 0.120 |
| WT2 | 47 | 0.120 | 0.108 |
| WT3 | 45 | 0.113 | 0.142 |
| WT4 | 54 | 0.130 | 0.136 |
| WT5 | 53 | 0.139 | 0.120 |
| Null 1 | 32 | 0.102 | 0.089 |
| Null 2 | 29 | 0.114 | 0.108 |
| Null 3 | 22 | 0.099 | 0.085 |
| Null 4 | 32 | 0.095 | 0.108 |
| Null 5 | 28 | 0.107 | 0.104 |
| WT Æ | 52 ± 5 | 0.130 ± 0.012 | 0.125 ± 0.012 |
| Null Æ | 24 ± 4 | 0.103 ± 0.007 | 0.099 ± 0.010 |

Note: Five 8-month-old male animals each (WT, *Csrp2*^-/-^) were comparatively analysed in cardiac echography. Abbreviations used are: FS, fractional shortening; WT, wildtype.
